# Dietary acid load decreases with age and is associated with sagittal abdominal diameter: a nationally representative quantification study in US adults

**DOI:** 10.1007/s40520-023-02508-6

**Published:** 2023-07-29

**Authors:** Maximilian Andreas Storz, Alvaro Luis Ronco

**Affiliations:** 1 Department of Internal Medicine II, Centre for Complementary Medicine, Freiburg University Hospital Faculty of Medicine, University of Freiburg, Freiburg, Germany; 2 Unit of Oncology and Radiotherapy, Pereira Rossell Women’s Hospital, Montevideo, Uruguay

**Keywords:** Dietary acid load, Nutrient intake, National Health and Nutrition Examination Surveys, Potential renal acid load, Anthropometric data, Net endogenous acid production

## Abstract

**Background:**

Dietary acid load (DAL) has been associated with frailty and hip fractures in older adults, who often have a reduced kidney function and thus compromised buffering capacities. Studies to quantify DAL in older adults are scarce and controversies persist as to whether DAL in- or decreases with age.

**Aim:**

To enhance the understanding of DAL in older individuals, we examined its relationship with increasing age and selected anthropometric data in a well-characterized sample of US adults.

**Methods:**

Secondary data analysis of nationally representative data from the National Health and Nutrition Examination Surveys data (NHANES 2011–2016). The sample included *n* = 3018 adults aged 60+, which may be extrapolated to represent *n* = 45,113,471 Americans. DAL was estimated using 4 formulas, including Potential Renal Acid Load (PRAL) and Net Endogenous Acid Production (NEAP).

**Results:**

All employed DAL scores tended to decline with increasing age. Participants aged 80 years or older yielded the lowest DAL scores. The average US citizen aged 60+ consumed an acidifying diet, yet there were sex-specific differences in the adjusted means for some scores. NEAP was positively correlated with both body mass index (*r* = 0.26, *p* < 0.001) and the sagittal abdominal diameter (*r* = 0.31, *p* < 0.001) in this nationally representative sample.

**Conclusion:**

The previously reported phenomenon of increasing DAL values in older people in non-Western countries may not apply to the US. Our findings may constitute an important step towards a better understanding of DAL in older US adults, and highlight the need for additional population-specific research in the field.

**Supplementary Information:**

The online version contains supplementary material available at 10.1007/s40520-023-02508-6.

## Introduction

Diet can essentially affect acid–base homeostasis in humans and may; thus, play an important role in health and disease [1]. Diets abundant in fruits and vegetables exert alkalizing effects on the human body due to their high content of base precursors [2, 3], whereas Western diets that focus on processed grains and animal products (including processed meats and cheese) have acidifying effects [4]. This is largely attributable to their high content of sulfur-containing amino acids, lysine, and arginine, which are major dietary acid precursors [5, 6]

The net balance between acidifying and alkalizing foods is called Dietary Acid Load (DAL) [7]. DAL may be assessed using nutrient- and anthropometric-based formulas, including Potential Renal Acid Load (PRAL) and Net Endogenous Acid Production (NEAP) [8]. The PRAL score is an established method to estimate the potential acid load of foods to the kidneys (in mEq/100 g edible food) and considers macro- and micronutrient intakes. In addition, the NEAP score also considers anthropometry-based estimates for organic acid excretion.

A high DAL has been linked to low-grade metabolic acidosis that may predispose individuals to metabolic imbalances and chronic non-communicable diseases including muscle mass loss, magnesium depletion, kidney disease, type-2-diabetes, hypertension and some cancers [9–13]. This may be of particular importance in older adults, as renal functional capacity and acid buffering abilities decline with aging [14]. A better renal function has been associated with higher availability of bases [14], which may help to buffer acids resulting from diet.

Whether DAL itself predisposes to kidney damage or whether a high net endogenous acid load is the consequence of impaired kidney buffer capacities is controversial. A Spanish cohort study revealed that participants with higher PRAL and NEAP scores had higher odds of a ≥ 10% estimated glomerular filtration rate decline in a prospective one year period [15]. The interplay between both factors is likely complex and not yet fully understood.

Yet, it is not surprising that a high DAL has been associated with unfavorable health outcomes in older adults. A high DAL has been linked to a higher prevalence of frailty (particularly in terms of slowness/weakness and low physical activity), hip fractures and impaired bone health, as well as metabolic syndrome [16–19].

Despite an increasing number of studies in recent years, many questions with regard to DAL in older adults remain essentially unanswered. Owing to the lower protein (and potassium) intake in older adults, PRAL and NEAP scores would theoretically supposed to be lower as compared to younger adults. This may be particularly true because DAL scores are not energy adjusted but reflect total nutrient intakes [8]. Yet, some authors reported higher DAL scores in older adults [20], thereby contradicting the expected findings based on the DAL formulas.

DAL reference ranges for older adults are still non-existent, particularly because many available studies were conducted in highly selective samples and populations. Yet it is clear that the typical DAL values of Western diets (ranging from 50 to 70 mEq/d 6) may not necessarily apply to older adults due to their specific and often hypocaloric nutrient intake profiles. To assess DAL values in a general population and to enhance our numeric understanding of DAL in older adults, new studies with nationally representative datasets are warranted.

We tried to fill this gap in the literature, using data from the US-Based National Health and Nutrition Examination Surveys. The aims of this study were twofold: I) to quantify DAL in the older US general population using nationally representative data, and II) to investigate whether the previously reported phenomenon of increasing DAL values in older adults may apply to the US, as well.

## Materials and methods

### Study population and design

We used aggregated population-based cross-sectional data from the National Health and Nutrition Examination Surveys (NHANES) to examine potential associations between DAL and age [21]. The NHANES is a US-specific annual program that collected data from the non-institutionalized population using a multistage stratified sampling technique to select participants [22]. Its hallmarks, the complex, stratified, multistage probability sampling design [23], allows the user to compute nationally representative health statistics.

Datasets from the NHANES are frequently used in age-related nutritional health research, and have been described elsewhere in detail [24, 25]. Briefly summarized, the NHANES was designed to assess the health and nutritional status of non-institutionalized adults in the United States [21]. It is one of several large health‐related programs conducted by the National Center for Health Statistics in the United States. NHANES includes clinical examinations, selected medical and laboratory tests, and self-reported data on nutrient intake and medical conditions [26]. Large parts of the data are freely available and accessible online. NHANES study and survey protocols were approved by the Research Ethics Review Board of the National Center for Health Statistics, and written informed consent was obtained from all participants [27]. All procedures and methods were carried out in full accordance with relevant guidelines and regulations.

### DAL assessment

DAL calculation methods have been described elsewhere [28, 30]. In the present analysis, we used 4 different DAL scores including: (I/II) Potential Renal Acid Load (PRAL_R_) and Net Endogenous Acid Production (NEAP_R_) as reported by Remer and Manz [31], (III) Net Endogenous Acid Production as reported by Frassetto et al. (NEAP_F_) [32], and (IV) Net Endogenous Acid Production as reported by Lemann and Kleinmann (NEAP_L_) [33]. All 4 scores are established in epidemiological and clinical research and demonstrated adequate performance in a head-to-head comparison. [29]

The PRAL_R_ score is a nutrient intake-based formula and was estimated as follows [34]:

$$\begin{gathered} {\text{PRAL}}_{{\text{R}}} \left( {{\text{mEq}}/{\text{day}}} \right) = \left( {0.{49} \times {\text{total protein }}\left( {{\text{g}}/{\text{day}}} \right)} \right) \hfill \\ + \left( {0.0{37} \times {\text{phosphorus }}\left( {{\text{mg}}/{\text{day}}} \right)} \right) \hfill \\ - \left( {0.0{21} \times {\text{potassium }}\left( {{\text{mg}}/{\text{day}}} \right)} \right) \hfill \\ - \left( {0.0{26} \times {\text{magnesium }}\left( {{\text{mg}}/{\text{day}}} \right)} \right) \hfill \\ - (0.0{13} \times {\text{calcium }}\left( {{\text{mg}}/{\text{day}}} \right)). \hfill \\ \end{gathered}$$

For NEAP_F_ we used the following formula [32]:

NEAP_F_ (mEq/d) = (54.4 × protein (g/day)/potassium (mEq/day)) − 10.2

To compute NEAP_R_, we used the following formula [34]:

$${\text{NEAP}}_{{\text{R}}} \left( {{\text{mEq}}/{\text{day}}} \right) = {\text{PRAL}}_{{\text{R}}} \left( {{\text{mEq}}/{\text{day}}} \right) + {\text{OAest }}({\text{mEq}}/{\text{day}}).$$

OA_est_ (mEq/d), an anthropometry‐based estimate for organic acid (OA) excretion which is also referred to as OA_anthr_ may be calculated as follows:

$${\text{Individual body surface area}} \times {41}/{1}.{73}.$$

NEAP_L_ was calculated as recently described by Parmenter et al. using the following Eq. 29:

$${\text{NEAP}}_{{\text{L}}} \left( {{\text{mEq}}/{\text{d}}} \right) = {\text{PRAL}}_{{\text{R}}} + {\text{OA}}_{{{\text{diet}}}} .$$

For OA_diet_, we used the following formula as described in [33, 34]:

$${\text{OA}}_{{{\text{diet}}}} \left( {{\text{mEq}}/{\text{day}}} \right) = {32}.{9} + (0.{15} \times \left[ {\left( {{\text{potassium }}\left( {{\text{mmol}}/{\text{day}}} \right)} \right) + \left( {{\text{calcium }}\left( {{\text{mmol}}/{\text{day}}} \right) \times {2}} \right) + \left( {{\text{magnesium }}\left( {{\text{mmol}}/{\text{day}}} \right) \times {2}} \right){-}\left( {{\text{phosphorus }}\left( {{\text{mmol}}/{\text{day}}} \right) \times {1}.{8}} \right)} \right]).$$

In summary, we calculated DAL based on the nutrient intake data and anthropometric data. Nutrient intake was obtained from the NHANES dietary data module, which includes nutritional assessments through two repeated 24-h dietary recalls. A full module description may be found elsewhere. [35]

### Age categories

Sociodemographic data and nutrient intake data as well as DAL scores were compared across 3 age categories following an approach by Tudor-Locke et al. [36] Age categories were as follows: (I) age 60–69, (II) age 70–79, and (III) age 80 years or older. In the NHANES, all responses of participants aged 80 years and older were coded as ‘80’. The reporting of age in single years for adults 80 years and older was determined to be a disclosure risk. As such, we were not able to compute the mean age and corresponding standard error in this age group. According to the NHANES, the weighted mean age for participants 80 years and older was 84 years in the NHANES 2013–2014 cycle and 85 years in the 2015–2016 cycle [37, 38].

### Body measures

Anthropometric data was obtained from the NHANES body measures module. Body Mass Index (BMI) was calculated as weight in kilograms divided by height in meters squared, and then rounded to one decimal place. In addition, we also included data on the average sagittal abdominal diameter (SAD) of each participant. The details on SAD measurements (including a graphical visualization) may be obtained from Li et al. [39]. In brief, SAD was measured by a trained examiner when the participant was in a supine position on an examination table. With the help of an abdominal caliper of proper size (Holtain Model 609XL, Seritex Inc., NJ, USA) examiners measured the external distance between the front of the abdomen and the small of the back at the iliac level line. Up to four SAD readings were taken following the above procedure. The SAD for each person was calculated as an average over the readings. The SAD is considered a simple and inexpensive anthropometric measure of visceral adiposity that has been linked to mortality and cardiovascular disease [39, 40]. According to Li et al., the SAD might be superior when compared to other anthropometric measures such as waist circumference, which does not distinguish visceral from subcutaneous adipose tissue. Owing to the relationship of some DAL scores with anthropometric data, 2 different anthropometric indicators were selected.

### Inclusion and exclusion criteria

For this analysis, we only considered individuals aged 60 years or older with available sociodemographic data, plausible energy intake data (based on the Willett’s criteria [41]) and DAL scores as well available anthropometric data. Individuals with an incomplete dataset (e.g. missing data on smoking status or other important variables) were excluded from the analysis.

### Statistical analysis

Data were analyzed using Stata 14 statistical software (StataCorp. 2015. Stata Statistical Software: Release 14. College Station, TX: StataCorp LP). In a first step, we appended 3 consecutive NHANES survey cycles (2011–2012, 2013–2014 and 2015–2016) to increase the sample size for analyses stratified by population subgroups.

Subsequently, subpopulation summary statistics and histograms were used to check for normality of the data. All continuous variables were normally distributed and described with their mean and standard error in parenthesis. For categorical variables, we presented weighted proportions and the corresponding standard error in parenthesis. The latter were estimated using Taylor series linearization to account for the complex NHANES sampling design.

To generate weighted percentages and means that are representative of the non-institutionalized civilian population, we used appropriate sample weights that also account for differential nonresponse and/or non-coverage, and which adjusted for planned oversampling of some groups. For this analysis, we constructed a 6-year-dietary data weight.

All weighted proportions were carefully checked for reliability as described previously [30, 42, 43]. Unreliable proportions were clearly flagged using superscript symbols. To test for potential associations between categorical sociodemographic variables and age category, we used Stata’s Rao–Scott test (a design-adjusted version of the Pearson chi-square test). Multivariate linear regression analyses (followed by adjusted Wald tests and Stata’s margins function) were used to test for potential differences in continuous variables between the 3 different age groups.

Apart from crude DAL scores, we also investigated adjusted DAL scores. For this, we used multivariate linear regression models in which we adjusted for the following covariates: age, sex, total energy intake and BMI. These parameters were previously shown to substantially influence DAL metrics [6, 44]. All multivariate linear regression models were constructed in line with the most recent recommendations of West, Berglund and Heeringa [45]. Post regression, we used Stata’s margins function and marginsplots to graph statistics from fitted models. We used a p value < 0.05 as a cutoff for statistical significance.

## Results

A total sample size of *n* = 3018 remained eligible for the final analysis after exclusion of those participants not meeting the inclusion criteria. This number may be extrapolated to represent *n* = 45,113,471 US American adults aged 60 years or older. Supplementary Fig. 1 shows the reasons for in- and exclusion of participants in a flow chart.

Sample characteristics are shown in Table 1. No significant differences were found with regard to the weighted proportions of men and females across the 3 examined age groups. Other sociodemographic characteristics (including race/ethnicity, educational level and marital status) differed significantly across the 3 age groups. The weighted proportion of overweight and obese individuals was lowest in participants aged 80 years or older, whereas it was highest in the group aged 60–69 years. A comparable distribution was found for smoking status, with the lowest rate of current smokers in individuals aged 80 years or older. All weighted proportions may be considered reliable as per the most recent NCHS guidelines, except the proportion of never married individuals in participants aged 80 years or older.

**Table 1:** Sample characteristics by age category

	60–69 years *n* = 1659	70–79 years *n* = 895	80 years or older *n* = 464	*p*-value
*Sex*				0.577^b^
Male Female	49.88% (1.51) 50.12% (1.51)	49.84% (2.02) 50.16% (2.02)	46.69% (2.45) 53.31% (2.45)	
*Age (years)*				< 0.001^c^
	64.17 (0.62)	73.70 (0.95)	80*	
*Marital status*				< 0.001^b^
Married/living with partner Widowed/divorced/separated Never married	76.29% (1.75) 19.33% (1.48) 4.38% (0.76)	72.52% (2.44) 25.36% (2.32)^e^ 2.12% (0.62)	54.56% (3.43)^e^ 42.63% (3.11)^e^ 2.81% (1.22)*	
*Race/ethnicity*				< 0.001^b^
Mexican American Other Hispanic Non-Hispanic White Non-Hispanic Black Other Race^a^	4.13% (0.71) 3.38% (0.57) 78.48% (1.90) 7.55% (1.08) 6.45% (1.09)	2.50% (0.51) 2.68% (0.50) 80.86% (2.16) 6.62% (1.08) 7.34% (1.23)	0.93% (0.29)^e^ 1.42% (0.52)^e^ 89.53% (1.66)^e^ 4.16% (0.81)^e^ 3.96% (1.14)	
Education level				0.002^b^
Less than 9th grade 9–11th grade High school graduate/GED^d^ Some college or AA degree College graduate or above	3.54% (0.61) 6.58% (0.91) 19.00% (1.42) 34.21% (2.22) 36.67% (2.42)	4.59% (0.65) 8.74% (1.21) 22.50% (2.00) 32.18% (2.11) 31.99% (2.73)	7.37% (0.97)^e^ 9.28% (1.42) 27.59% (2.66)^e^ 29.41% (2.39) 26.35% (2.30)^e^	
*BMI*				< 0.001^a^
< 18.50 ≥ 18.50 and < 25.00 ≥ 25.00 and < 30.00 ≥ 30	0.47% (0.19) 22.60% (2.02) 35.53% (1.77) 41.41% (2.24)	1.36% (0.59) 25.46% (1.74) 37.28% (1.96) 35.89% (2.54)^e^	1.52% (0.47) 34.21% (2.63)^e^ 42.60% (2.10)^e^ 21.67% (1.83)^e^	
*Smoking status*				< 0.001^b^
Non-smoker Current smoker Former smoker	50.04% (2.06) 12.34% (1.01) 37.62% (1.73)	48.54% (1.96) 7.42% (1.18)^e^ 44.04% (1.93)^e^	50.98% (2.92) 2.18% (0.76)^e^ 46.84% (2.73)^e^	

Weighted proportions. Total number of unweighted observations: 3018. Continuous variables shown as mean (standard error). Categorical variables shown as weighted proportion (standard error). All weighted proportions can be considered reliable, as peer recent NCHS Guidelines, except for those marked with an “*”. a = includes multi-racial; b = based on the Stata’s design-adjusted Rao–Scott test, c = based on the regression analyses followed by adjusted Wald tests, d = or equivalent, e = indicates significant differences in the weighted proportions. Columns may not equal 100% due to rounding

Table 2 shows energy and nutrient intake across the 3 age groups. Higher age was inversely associated with energy intake (Table 2), whereas no significant differences were found with regard to fiber intake. We also observed significant differences in the mean intakes of several DAL-relevant nutrients, including protein (*p* < 0.001), phosphorus (*p* = 0.002) and magnesium (*p* = 0.011). No differences were observed with regard to the daily intake of potassium and calcium.

**Table 2:** Energy and nutrient intake by age category

	60–69 years *n* = 1659	70–79 years *n* = 895	80 years or older *n* = 464	*p*-value
Energy intake (kcal/day)	2004.01 (26.19)	1898.24 (26.59)	1826.45 (27.74)	< 0.001
Carbohydrate intake (g/day)	235.38 (3.96)	225.45 (4.27)	225.82 (4.17)	0.096
Fat intake (g/day)	80.30 (1.19)	75.54 (1.34)	71.84 (1.49)	< 0.001
Protein intake (g/day)	78.09 (1.08)	74.56 (1.09)	67.76 (1.52)	< 0.001
Fiber intake (g/day)	17.95 (0.42)	17.95 (0.43)	16.88 (0.53)	0.306
Potassium intake (mg/day)	2755.29 (33.93)	2678.99 (48.61)	2645.78 (66.48)	0.233
Sodium intake (mg/day)	3344.18 (57.56)	3160.84 (56.25)	2872.61 (58.75)	< 0.001
Phosphorus intake (mg/day)	1326.53 (19.62)	1280.40 (20.60)	1210.67 (28.05)	0.002
Calcium intake (mg/day)	907.63 (20.95)	887.54 (21.74)	875.079 (27.61)	0.559
Magnesium intake (mg/day)	308.25 (5.80)	289.78 (5.36)	283.09 (6.97)	0.011

Total number of unweighted observations: 3018. Continuous variables shown as mean (standard error). All *p*-value s are based on the regression analyses followed by adjusted Wald tests

Table 3 displays crude DAL scores by age category. We observed significant intergroup differences for all 4 DAL estimates, including PRAL_R_, NEAP_R_, NEAP_F_, and NEAP_L_. DAL scores decreased with increasing age and were lowest in participants aged 80 years or older.

**Table 3:** Crude DAL scores by age category

	60–69 years *n* = 1659	70–79 years *n* = 895	80 years or older *n* = 464	*p*-value
PRAL_R_	9.67 (0.83)	8.58 (0.89)	3.70 (1.09)	< 0.001^a^
NEAP_R_	55.39 (0.99)	53.19 (0.93)	45.70 (1.21)	< 0.001^a^
NEAP_F_	52.55 (0.73)	51.46 (0.97)	46.24 (1.25)	< 0.001^a^
NEAP_L_	52.21 (0.75)	50.85 (0.78)	46.27 (0.96)	< 0.001^a^
OA_anthr_	45.72 (0.28)	44.61 (0.22)	42.00 (0.30)	< 0.001^a^
OA_diet_	42.54 (0.19)	42.27 (0.22)	42.57 (0.28)	0.486

Total number of unweighted observations: 3018. Continuous variables shown as mean (standard error). a = based on the regression analyses followed by adjusted Wald tests

PRAL_R_ = potential renal acid load—based on the formula by Remer et al. [34]; NEAP_R_ = net endogenous acid production—based on the formula by Remer et al. [34]; NEAP_F_ = net endogenous acid production—based on the formula by Frassetto et al. [32]; NEAP_L_ = net endogenous acid production—based on the formula by Lemann and Kleinmann [33]; OA_anthr_ = anthropometry‐based estimate for organic acid (OA) excretion; OA_diet_ = dietary‐based estimate for organic acid (OA) excretion

**Fig. 1:**
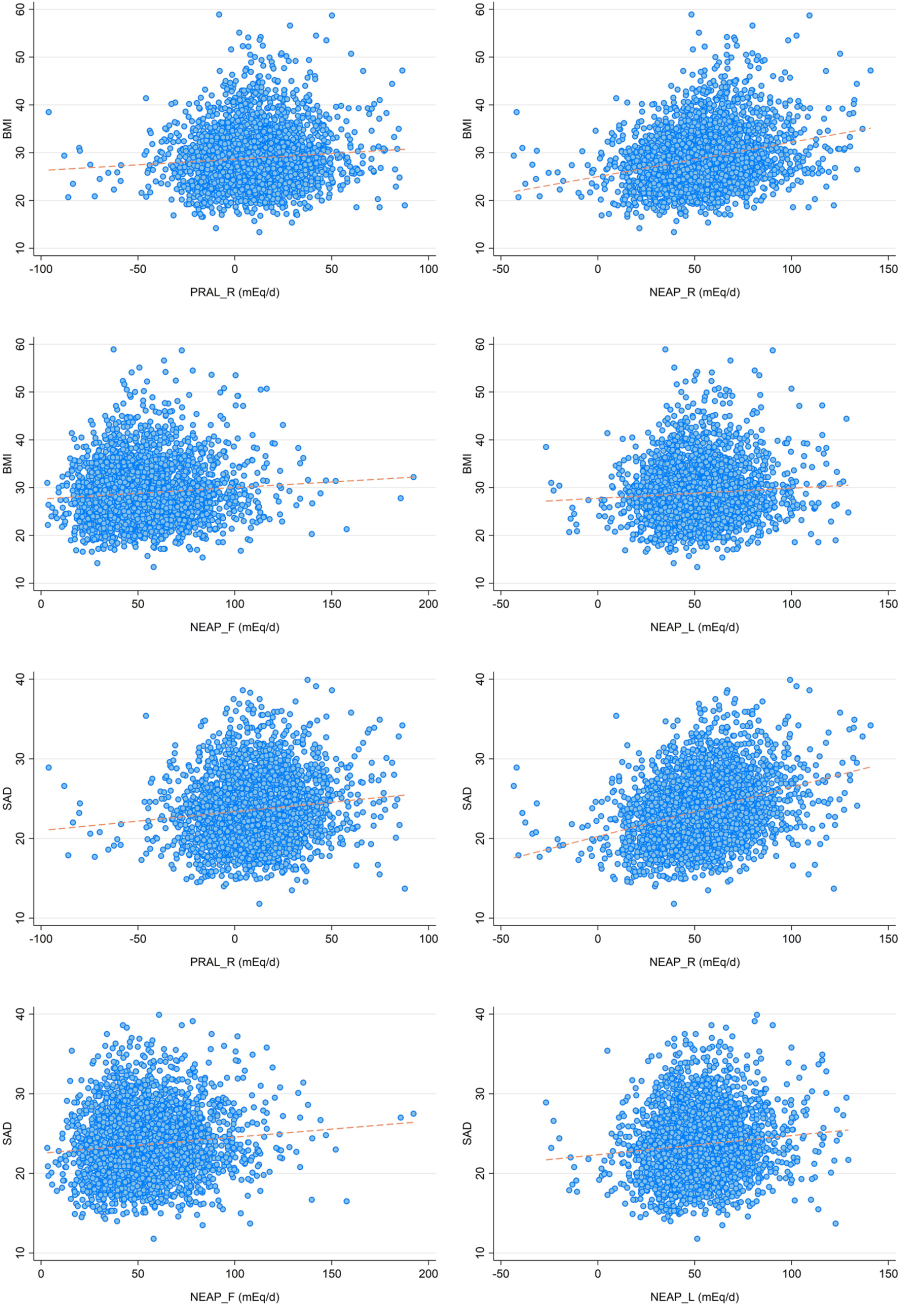
The relationship between DAL estimates and anthropometric measures. Scatterplots of BMI (or SAD) and all 4 DAL estimates (entire sample); including PRAL_R_ (top left), NEAP_R_ (top right), NEAP_F_ (bottom left), and NEAP_L_ (bottom right). BMI = Body Mass Index. SAD = sagittal abdominal diameter

We ran a Pearson's product‐moment correlation to assess the relationship between DAL scores and anthropometric data in all participants (see Fig. 1). Results suggested a small to moderate positive correlation between the NEAP_R_ score and BMI (*r* = 0.26, *p* < 0.001) as well as with the sagittal abdominal diameter (*r* = 0.31, *p* < 0.001). Correlation coefficients for the other DAL scores and anthropometric measures were all below *r* = 0.15 but significant as well (*p* < 0.05).

**Fig. 2:**
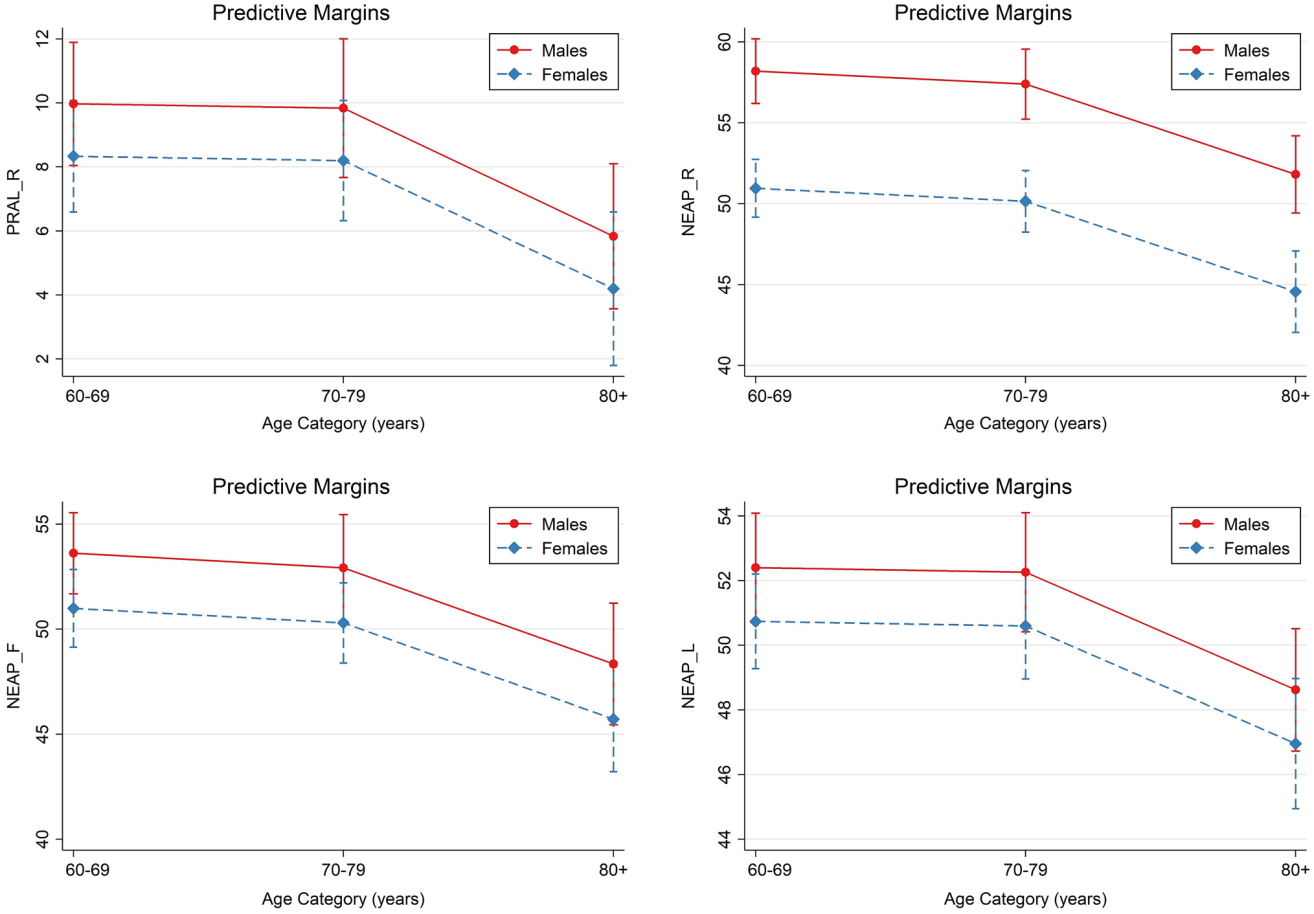
Margins plots—predicted DAL estimates by age category. Plots of marginal predicted values based on the multivariate regression models adjusting for race/ethnicity, sex, age, BMI and total energy intake. The plots illustrate differences in the relationship of each DAL estimate and age category, depending on sex. DAL estimates shown include PRAL_R_ (top left), NEAP_R_ (top right), NEAP_F_ (bottom left), and NEAP_L_ (bottom right)

Multivariate linear regression models were run to examine the associations between DAL scores and age after adjustment for covariates (including sex, BMI and total energy intake). Based on these models (not shown), sex-specific marginal predicted values for all age groups are shown in Fig. 2. Participants aged 80 years or older yielded substantially lower DAL scores than participants aged 60–69 years, suggesting a potential DAL decrease with increasing age. In some occasions, those differences were statistically significant (e.g. for NEAP_R_). Females yielded lower DAL scores than men with regard to all 4 scores. However, significant differences between both sexes were only found for PRAL_R_.

## Discussion

The present study quantified DAL in the older US general population and thereby revealed some noteworthy trends: (I) All employed DAL scores (PRAL_R_, NEAP_R_, NEAP_F_ and NEAP_L_) tended to decline over the investigated age categories, (II) individuals aged 80 years or older yielded the lowest DAL scores, (III) the average person aged 60 + in the US consumed an acidifying diet (as reflected by positive PRAL sums), and (IV) the previously reported phenomenon of increasing DAL values in older people in non-Western countries may not apply to the US.

DAL may play an important role in health and disease, as largely acidifying diets have been linked to several chronic health conditions [28]. DAL may be of particular importance in older individuals in whom kidney function naturally declines [46]. This, in turn, may impair the body’s capacity to buffer acids resulting from diet [14]. Notably, the relationship between DAL and health is often examined in young individuals under metabolic ward conditions. Studies in free living adults are limited and often stem from various different populations and ethnicities [20].

Kataya et al. reported that DAL was positively associated with the prevalence of frailty and particularly slowness/weakness as well as low physical activity in older Japanese women [16]. The authors investigated a sample of 2176 Japanese women aged 65–94 years. The mean PRAL_R_ scores in this cohort was 4.9 ± 12.3 mEq/d and differed substantially between frail (4.7 ± 12.6 mEq/d) and non-frail women (6.2 ± 10.7). Minimal at a first glance, such PRAL_R_ differences may have a large clinical impact when sustained over a long time as reflected by a recent large-scale Chinese case–control study by Li et al. [17]. The authors investigated whether DAL was associated with the risk of hip fractures among middle-aged and older people in the Guangdong Province, in China. Again, differences in between cases (21.2 (16.7, 24.9) mEq/d) and controls (18.3 (12.9, 23.2) mEq/d) were small but statistically significant.

Of note, these examples highlight the difficulties in cross-country and population comparisons of DAL. DAL reference ranges and large population-based analyses in this field are rare—and the majority of analyses do not stem from nationally representative data but from very selective samples. What may be considered “a high DAL” is currently subject to a controversial debate. We aimed to address this point with our current submission and estimated nationally representative adjusted mean DAL values for the US.

Thereby, we encountered an opposite trend in comparison to the previous findings by Alam et al. [20], who examined a healthy, free-living elderly population in Pakistan. Their sample comprised 526 older and 131 young participants (aged 50–80 and 23–28 years, respectively), which yielded significant differences in their DAL scores. Notably, those aged 50–80 years (mean age 69.1 ± 5.6 years) had higher DAL scores (3.33 ± 0.8 mEq/d) as opposed to the younger generations with a mean age of 23.5 years (1.2 ± 1.2 mEq/d). Alam et al. reported that older people in their study had a tendency to eat vegetables (including potatoes, green leafy vegetables, pumpkin, etc.), which are alkali-generating, only when cooked. In addition, they ate these foods only in conjunction with meat, fish, and eggs, which may be one of the reasons for the increased dietary acidity, whereas the young participants ate vegetables alone, which were mostly uncooked, in the form of salad, for example. This may constitute a unique dietary pattern in the elderly study population of this particular sample and could be a special case with regard to DAL calculations. In general, though, when glancing at the DAL calculation methods, it is to be expected that DAL decreases with older age (largely attributable to the lower total energy and protein intake). Our analysis confirms this trend for the US and is in line with other previous studies in Western societies [47, 48].

On the other hand, our results could generate the erroneous impression of “safety”. The decrease of DAL with advancing age does not imply that the body is automatically exposed to a lower acid burden from diet. To the contrary, renal function (and thus buffering capacities) declines with age, as well [14]. Frassetto and Sebastian reported that with increasing age, there is a significant increase in the steady-state blood [H^+^] (*p* < 0001), and reduction in steady-state plasma [HCO_3_^−^] (*p* < 0.001), indicative of a progressively worsening low-level metabolic acidosis [49]. Diet alone may not be the decisive factor here, and must always be considered in conjunction with renal function to assess acid-related damage in older populations.

While our results do not help to understand this complex interplay, our data suggests that there is room for potential with regard to lowering the acid-forming potential of diets in older US adults. The average American diet in this sample was acidifying, and targeted strategies focusing on an increased intake of high quality plant-protein low in acid precursors (e.g. legumes, nuts, seeds) could help counteracting this phenomenon.

The associations between the NEAP_R_ score and the SAD in our sample are also noteworthy, because the latter was identified as an independent predictor of all-cause and cardiovascular mortality in various studies [50, 51]. Paying increasing attention to DAL in older individuals could thus be beneficial from multiple perspectives. A first step to achieve this goal is a detailed quantification of DAL in a nationally representative sample such as the NHANES—as it was performed in our study.

Apart from this apparent strength, our analysis has also weaknesses to consider. The quantification-based character without adding disease-specific outcomes may appear pale at a first glance and does not contribute to a better understanding of DAL in disease and unfavorable metabolic conditions. On the other hand, we aimed at a high number of unweighted observations when merging the datasets in order to analyze a sample that may be extrapolated to represent a number of US adults as large as possible and to capture a picture “as broad as possible”. The non-inclusion of kidney function parameters also decreases the value of this analysis, although it is widely known that glomerular filtration rate decreases with age. Food group-based recommendations to decrease DAL in older individuals (without compromising overall quality protein intake) would have also enhanced this study but were too far outside the scope and will be presented in a separate analysis in the near future.

## Conclusions

DAL as assessed by four different scores (PRAL_R_, NEAP_R_, NEAP_F_ and NEAP_L_) in US adults appears to decline with increasing age in a nationally representative US sample of people aged 60+ years. The previously reported phenomenon of increasing DAL values in older people in non-Western countries may not apply to the US. Older adults in the US generally consume an acidifying diet and the association of NEAP_R_ with SAD warrants caution and—in line with the previous evidence—calls for new strategies to lower DAL in US adults. Our findings may constitute an important step towards a better understanding of potential reference values for different DAL scores in US adults and highlight the need for additional research in the field of acid–base balance in health and disease.

### Supplementary Information

Below is the link to the electronic supplementary material.

Supplementary Fig. 1 title: Participant inclusion flowchart. Supplementary Fig. 1 legend: Participant inclusion flowchart with reasons for in- and exclusion. n = 6 participants were excluded for implausible DAL scores (PNG 128 KB)

## Data Availability

Data is publicly available online (https://wwwn.cdc.gov/nchs/nhanes/Default.aspx). The datasets used and analyzed during the current study are available from the corresponding author on reasonable request.
