# Supplementary figures and images for: Dietary acid load decreases with age and is associated with sagittal abdominal diameter: a nationally representative quantification study in US adults
Source: Aging Clin Exp Res. 2023 Jul 29;35(10):2191–200. doi: 10.1007/s40520-023-02508-6 (PMC10519865; doi:10.1007/s40520-023-02508-6)

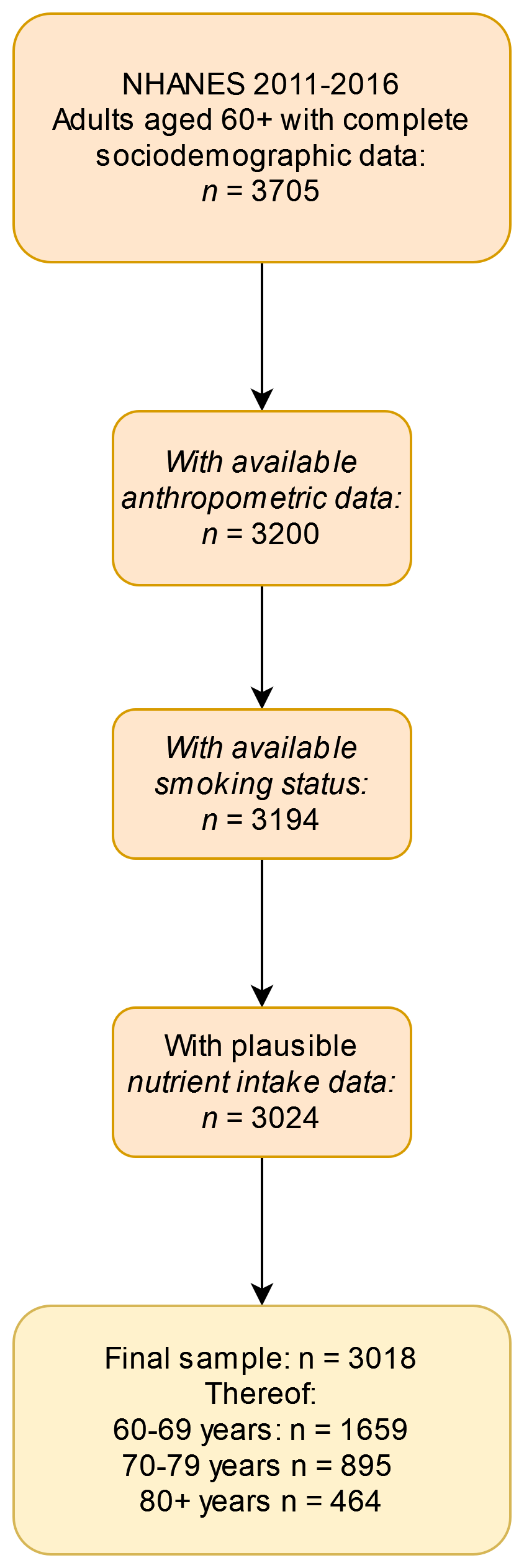

Supplement: Supplementary file 1 — Supplementary Fig. 1 title: Participant inclusion flowchart. Supplementary Fig. 1 legend: Participant inclusion flowchart with reasons for in- and exclusion. n = 6 participants were excluded for implausible DAL scores (PNG 128 KB) [file 40520_2023_2508_MOESM1_ESM.png]
